# Changes in Histamine Receptors (H1, H2, and H3) Expression in Rat Medial Vestibular Nucleus and Flocculus after Unilateral Labyrinthectomy: Histamine Receptors in Vestibular Compensation

**DOI:** 10.1371/journal.pone.0066684

**Published:** 2013-06-19

**Authors:** Liuqing Zhou, Wen Zhou, Sulin Zhang, Bo Liu, Yangming Leng, Renhong Zhou, Weijia Kong

**Affiliations:** Department of Otorhinolaryngology, Union Hospital of Tongji Medical College, Huazhong University of Science and Technology, Wuhan, Hubei province, P. R. China; Tokyo Medical and Dental University, Japan

## Abstract

Vestibular compensation is the process of behavioral recovery following peripheral vestibular lesion. In clinics, the histaminergic medicine is the most widely prescribed for the treatment of vertigo and motion sickness, however, the molecular mechanisms by which histamine modulates vestibular function remain unclear. During recovery from the lesion, the modulation of histamine receptors in the medial vestibular nucleus (MVN) and the flocculus may play an important role. Here with the means of quantitative real-time PCR, western blotting and immunohistochemistry, we studied the expression of histamine receptors (H1, H2, and H3) in the bilateral MVN and the flocculus of rats on the 1st, 3rd, and 7th day following unilateral labyrinthectomy (UL). Our results have shown that on the ipsi-lesional flocculus the H1, H2 and H3 receptors mRNA and the protein increased significantly on the 1st and 3rd day, with compare of sham controls and as well the contralateral side of UL. However, on the 7th day after UL, this expression returned to basal levels. Furthermore, elevated mRNA and protein levels of H1, H2 and H3 receptors were observed in the ipsi-lesional MVN on the 1st day after UL compared with sham controls and as well the contralateral side of UL. However, this asymmetric expression was absent by the 3rd post-UL. Our findings suggest that the upregulation of histamine receptors in the MVN and the flocculus may contribute to rebalancing the spontaneous discharge in bilateral MVN neurons during vestibular compensation.

## Introduction

Unilateral labyrinthectomy (UL) induces characteristic postural and oculomotor asymmetry in rodents, many of the deficits subside within a week after UL, the so-called period of adaptive plasticity, which is described as vestibular compensation. [1] The neurophysiological basis of vestibular compensation is the asymmetrical mass discharge on bilateral neurons in vestibular nuclear complex after UL. [2], [3] However, it is still not clear about the molecular mechanisms of vestibular compensation. Because UL induced a permanent destroy of vestibular end organ, the compensatory process has been attributed to central nervous system plasticity. [4], [5] Many regions of the brain such as the medial vestibular nucleus (MVN) and the flocculus have been shown to be involved in the process of vestibular compensation [6].

In the process of vestibular compensation, it has been suggested that the flocculus plays a crucial role. For instance, in behaviour research it has been shown that vestibular compensation may be delayed dramatically by two ways. [2], [7] The one is ablation of the vestibule-cerebellum, particularly paraflocculus-flocculus, uvula and nodulus, and the other is disruption of climbing fiber inputs to the flocculus. Even after compensation, flocculectomy leads to the reappearance of UL-induced behavioral deficits. [8] Recently, some neuroanatomical data [9], [10] have indicated that the floccular target neurons are localized to the rostral MVN, and these neurons receive monosynaptic inhabitation from the flocculus. In electrophysiological experiments, it has been reported that both the inhibitory signals from the cerebellar Purkinje cells and the commissural inhibition system mediate the silencing of ipsi-lesional MVN neurons after UL. [8] Hence, the MVN and flocculus may be the both important areas in vestibular compensation, although the neurochemical mechanism involved in this process remains largely unrevealed.

Previous studies have indicated that many neurotransmitters, such as Gamma-amino-butyric acid (GABA), glutamate, glycine, histamine and so on are involved in the process of vestibular compensation. [11] Histamine acts mainly as a neuromodulator rather than a neurotransmitter in the central nervous system. [12], [13] Furthermore, H3 receptor mediate presynaptic inhibition of releasing other neurotransmitters including: noradrenaline, serotonin, dopamine, glutamate, and GABA. [14], [15] All of the above neurotransmitters are implicated in central vestibular neurotransmission. [16] In clinics, the histaminergic medicine is the most widely prescribed for the treatment of vertigo and motion sickness, although the molecular mechanisms by which histamine modulates vestibular function remain unclear. [17], [18] In behaviour research of cats, it has been shown that betahistine and thioperamide both strongly accelerated behavioral recovery following UL. The first one is an H3 receptor antagonist and a weak H1 receptor agonist, and the later one is a more selective H3 receptor antagonist, the influence on behaviour may be due to an increased release of histamine in the brain. [19] Some neuroanatomical studies have shown that the soma of histaminergic neurons are located in the tuberomammillary nucleus of the posterior hypothalamic region, these histaminergic neurons send axonal projections to the whole vestibular nuclei complex and cerebellum. [20], [21] Different histamine receptors distribute differently on the neurons of the MVN and the flocculus. The H1 and H2 receptors are located postsynaptically, while the H3 receptor is located presynaptically. It has been shown that the H3 autoreceptor is located on the afferent histaminergic fibers of the neurons in the MVN and the flocculus locates. The H1, H2 and H3 heteroreceptors may be located either on the perykaria [22] or on the non-histaminergic afferents fibers of the neurons in the MVN and the flocculus. [1], [22]–[24] Functionally, it has been reported that the histamine receptors have an excitatory effect on both the MVN and the cerebellar neurons. [23]–[26] Taken together, the histamine receptors in the MVN and the flocculus may play a crucial role in vestibular compensation.

However, previous studies in different species have provided inconsistent data on histamine receptors mRNA and protein expression in the MVN during vestibular compensation. For instance, Tighilet *et al* [27] reported that the levels of H3 receptor binding were downregulated in the MVN of cats following unilateral vestibular neurectomy. On the contrary, Lozada *et al* [28] found H3 receptor mRNA to be upregulated in the MVN of rats after UL. Therefore, it remains unclear how the histamine receptors are expressed indeed with vestibular compensation. How do the expression of H1, H2 and H3 receptors mRNA and the corresponding protein levels of the MVN and flocculus change after UL (for example, on the 1st, the 3rd and the 7th day)? Is the performance of gene expression activities in the ipsi-lesional MVN and flocculus different from that in the contra-lesional side, and from sham control as well? All these questions will be answered in the end of our studies.

## Materials and Methods

### Ethics Statement

This study was carried out in strict accordance with the recommendations in the Guide for the Care and Use of Laboratory Animals of the National Institutes of Health. The protocol was approved by the Committee on the Ethics of Animal Experiments of the University of Huazhong University of Science and Technology (Permit Number: S251). All surgery was performed under a mixture of ketamine and chlorpromazine anesthesia, and all efforts were made to minimize suffering.

### Animal Model

Adult male Sprague-Dawley rats (200–250 g) were obtained from the experimental animal center of Tongji Medical College, Huazhong University of Science and Technology. In despite of poor visual acuity, [29], [30] the albino rats have well gained rebalance after UL, so vestibular compensation process of the albino rats is reliable and complete. Hence, the unilateral labyrinthectomy on albino rats is a mature and generally accepted animal model in vestibular compensation. Furthermore, some researches indicated that the albino rats strain (Sprague-Dawley rats) has better auditory acuity and excelling in skilled reaching compared to other rat strains. [31], [32] The animals had free access to food and water. A total number of 90 rats were divided into three groups, as follows: 36 rats for quantitative real-time PCR, 36 rats for western blotting and 18 rats for immunohistochemistry. Experimental animals in each group were randomly divided into sham controls and experimental groups on the 1st, 3rd and 7th day after UL. The post-surgery times were chosen based on previous reports in rats. [2], [33] The 1st day after UL represents the acute, uncompensated stage when spontaneous nystagmus is vigorous and postural asymmetry is severe. By the 3rd day, these symptoms have partly diminished, and by the 7th day, the static symptoms have substantially compensated but dynamic reflex deficits remain. After decapitation under anesthesia (ketamine-chlorpromazine mixture 10∶1, 2 ml/kg, intraperitoneal injection), a complete surgery was performed on the right side under an operating microscope according to the method reported by Kitahara *et al*. [34] Firstly, the tympanic bulla was exposed by a retroauricular approach and opened using the surgical electrodrill, and then the stapedial artery was exposed for coagulation. Secondly, after a small hole was made around the oval window using the surgical electrodrill, the membranous labyrinth was removed and aspirated with a suction pump. Thirdly, absolute alcohol was injected into the opening to ensure that the vestibule was completely destroyed. At the end of surgery, the wound was sutured. For a control of confounded effects of anesthesia and unilateral soft tissue injury, rats were submitted to a sham operation under the same anesthesia, the tympanic bulla on the right side was opened, but the membranous labyrinth was not destroyed before suture. It has been reported that alcohol can influence the histamine level in some regions of the brain by alcohol consumption (drinking) both in rodents and in humans. [35], [36] However, the change patterns of histamine receptors expression levels after UL using only the mechanical UL method in our preliminary experiment were well coincident with that by a combined UL method (Fig. S1).

### Quantitative Real-time PCR

Quantitative analysis of the H1, H2 and H3 receptors mRNA in the MVN and the flocculus was performed by real-time PCR. On the 1st, 3rd and 7th day after UL or sham operation, 36 rats (n = 6 per group) were killed by cervical dislocation without anesthesia. Following the rat brain atlas of Paxinos and Watson, [37] the bilateral flocculus was carefully removed from each brain and the bilateral MVN was dissected under a microscopic using the detailed procedures as described previously by Horii *et al*. [6] Total RNA was extracted using an RNeasy mini kit (Axygen, USA) according to the manufacturer’s instructions. cDNA was reverse transcribed using a PrimeScript RT reagent kit with gDNA Eraser (TaKaRa, Japan). The RNA and cDNA of each sample were analysed using a GeneQuant pro RNA Calculator to assess the concentrations and purity. Quantitative real-time PCR was performed with real-time SYBR Green PCR reagents and the 7300 Real-Time PCR System (Applied Biosystems, Foster City, CA). Validated primers were designed for each target mRNA. The primer pairs for H1, H2, H3 and an internal standard (β-actin) were as follows: H1 forward, 5′-CTGGTCACAGTGGGCCTCAA-3′; H1 reverse, 5′-CTGCCACAGACAGGCT-GACAA-3′; H2 forward, 5′-ATGGAGCCCAATGGCACAG-3′; H2 reverse, 5′-GCCAGCAATGGTGATGAGGA-3′; H3 forward, 5′-CCTCGGTCTTCAACATC-GTACTCA-3′; H3 reverse, 5′-ACCCACACC ATGCCATCTTC-3′; β-actin forward, 5′-CCTGGAGAAGAGCTATGAGC-3′; β-actin reverse, 5′-ACAGGATTCCATACC-CAGG-3′. The amplification conditions were as follows: 1 min at 95°C, and then 40 cycles of 15 s at 95°C, 20 s at 60°C, and 35 s at 72°C. An internal standard was used to normalize the relative gene expression levels. A melting curve analysis was performed for each gene, and the specificity and integrity of the PCR products were confirmed by the presence of a single peak. The relative expression values were calculated from the differences in Ct of the values between the target mRNA and an internal standard (β-actin). The change in the corresponding mRNA levels between the experimental group and the control group was analyzed with the 2^−ΔΔCt^ method, as the previous literature reported [38].

### Western Blotting

The aim of this experiment is to quantify the protein expression of H1, H2 and H3 receptors in the MVN and the flocculus. Animals were selected on the 1st, 3rd and 7th day after UL, and similarly with sham operation as well. Totally 36 rats (n = 6 per group) were executed by cervical dislocation without anaesthesia. Following the rat brain atlas of Paxinos and Watson, [37] the bilateral flocculus was carefully removed from each brain and the bilateral MVN was dissected under a microscopic using the detailed procedures as described previously by Horii *et al*. [6] The total protein in the MVN and the flocculus was extracted using a RIPA Lysis Buffer (Beyotime, Haimen, China) according to the manufacturer’s instructions. Protein concentrations were determined with an Enhanced BCA Protein Assay Kit (Beyotime, Haimen, China). Twenty micrograms of each protein lysate was separated by 8% SDS-polyacrylamide gels and transferred to polyvinylidene difluoride (PVDF) membranes. The membranes were incubated for 1 hour in a blocking solution of TBS containing 5% non-fat milk, washed briefly in TBS and incubated overnight at 4°C with the appropriate dilution of the following primary antibodies: anti-H1 (Santa Cruz, USA, diluted 1∶500), anti-H2 (Santa Cruz, USA, diluted 1∶250) or anti-H3 antibody (Santa Cruz, USA, diluted 1∶500). After washing the membranes to remove excess primary antibody, the membranes were incubated for 1 h at room temperature with the appropriate horseradish peroxidase (HRP)-conjugated secondary antibody (Beyotime, Haimen, China, diluted 1∶5000). Membranes were visualized with BeyoECL Plus (Beyotime, Haimen, China). Quantitation of the detected bands was performed with the Image-Pro Plus 6.0 software (Media Cybernetics, Inc., USA). β-actin was used as an internal control.

### Immunohistochemistry

Following the completion of the quantitative real-time PCR and western blotting studies, immunohistochemistry was used to label and describe the distribution of the H1, H2 and H3 receptors on neurons of the flocculus and the MVN. On the 1st, 3rd and 7th day after UL or sham operation, 18 rats (n = 3 per group) were anesthetized deeply with a ketamine-chlorpromazine mixture (10∶1, 2 ml/kg, intraperitoneal injection), and perfused transcardially with a 0.9% normal saline wash followed by fixative (4% paraformaldehyde in 0.1 M phosphate buffer). After decapitating the bodies, the temporal bones were removed, and the brains were dissected out. The cerebellum and brain stem portion were fixed in the paraformaldehyde-phosphate buffer for 12 hours at 4°C, rinsed with distilled water for half an hour, dehydrated with a graded alcohol series, cleared in xylene, immersed in paraffin, and then embedded in paraffin. Finally, brainstems with flocculus were sectioned at 5 µm and collected on poly-L-lysine-coated glass slides.

After the sections were deparaffinized, they were incubated overnight at 4°C in anti-H1, anti-H2 or anti-H3 antibody (Santa Cruz, USA, diluted 1∶100). The sections were washed and incubated in a goat anti-rabbit secondary antibody (Beyotime, Haimen, China) for 60 minutes at room temperature. The nuclei were counter-stained with a DAPI staining solution (Beyotime, Haimen, China) for 5 min at room temperature. After washing with PBS, the sections were examined under a laser scanning confocal microscope (Nikon, Japan).

### Neurons Counting

Three animals from each group were used for neurons counting analysis. Four sections from the rostral to the caudal level of MVN or flocculus (Bregma −9.96 mm - Bregma −12.00 mm) in each rat were selected for counting the H1, H2 and H3 receptors-positive neurons following the guidance of the atlas [37]. Two sections were selected from the rostral and the caudal part of the brain [39] (Bregma −9.96 mm - Bregma −12.00 mm) including the MVN or ﬂocculus using the guidance of the atlas. [37] They represented the rostral and caudal zones of Purkinje cell/climbing fiber zones, which control the eye movement in all vertical planes from sagittal to transverse planes. Two sections at 50 µm intervals were selected from the middle part of the brain [40] (Bregma −9.96 mm - Bregma −12.00 mm) including the MVN or ﬂocculus using the guidance of the atlas. [37] They represented the middle zone of Purkinje cell/climbing fiber zones, which controls the eye movement in the horizontal plane [41], [42].

The number of H1, H2 and H3 receptors-positive neurons was counted in each corresponding region of the visual system in the same area of the bilateral flocculus and the bilateral MVN, with the number of labeled neurons expressed per unit area (mm^2^). [43], [44] Neurons were counted only if their nuclei were completely within the margins of the visual system by using Image-Pro-Plus (6.0) software (Media Cybernetics, Inc., USA). All images were acquired using the same exposure time and illumination conditions. Histological quantification was conducted by an investigator blinded to the experimental status of the animals.

### Statistical Analysis

Data are presented as means±SEM. The analysis was performed with SPSS 13.0 software (SPSS Inc., USA). All data were analyzed using one-way analysis of variance (ANOVA) followed by student Newman-Keuls multiple comparison tests. ANOVAs were performed only on data that were obtained from the same gels and 96-well plates. Because we ran tissue from the ipsi-lesional, sham and contra-lesional conditions on the one set of gels and 96-well plates, data were analysed separately for the 1st day, 3rd day and 7th day condition. This meant that comparisons between the ipsi-lesional, sham and contra-lesional conditions were not confounded by changes in the background density between different gels and 96-well plates. The significance level was set at 0.05 for all comparisons.

## Results

The results are analysed and presented from three different aspects. 1) By real-time PCR we can analyse quantitatively the H1, H2 and H3 receptors mRNA expression in different stage after UL. 2) The corresponding H1, H2 and H3 receptors protein expression is quantified with the means of western blotting. 3) The location and distribution of H1, H2 and H3 receptors is analysed by immunostaining of the brain sections, particularly the MVN and the flocculus.

### Behavior Observation after UL

**Figure 1:**
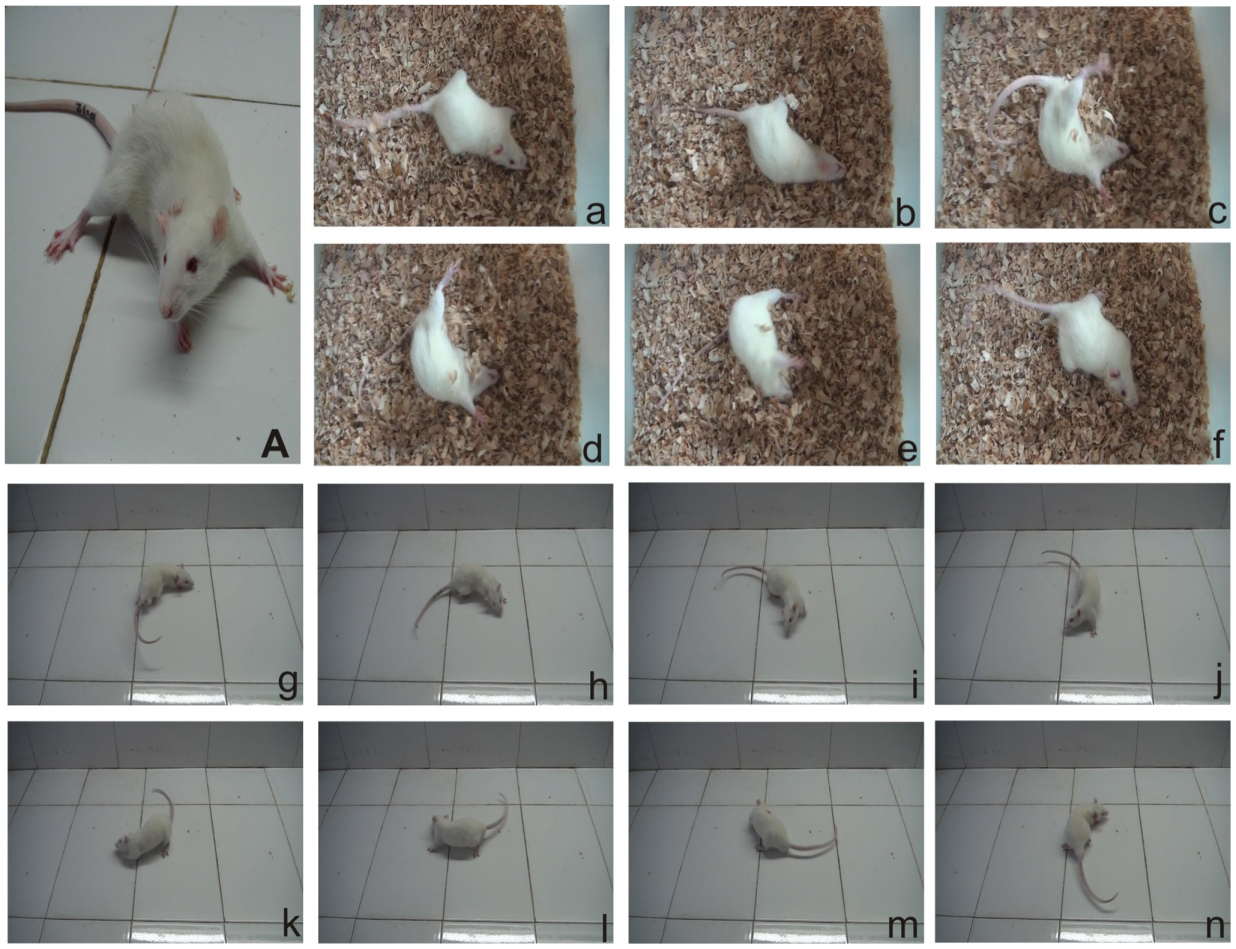
Behavior observation after UL. Behavioral asymmetries after UL on the right side. (A) The animal laid on the lesioned (right) side and showed hypotonia in the ipsilateral limbs, while the contralateral limbs were hypertonic, the head tilting towards to the operated side. (a–f) and (g–n) show the barrel rolling and circling walk, respectively.

Immediately after recovery from anaesthesia, rats that underwent UL manifested diverse behavioural symptoms: head tilting toward the operated side, limb extending on the intact side (Fig. 1A), barrel rolling (Fig. 1a–f), circling walk (Fig. 1g–n) and spontaneous nystagmus. These symptoms largely disappeared with the 1st day to the 7th day after UL.

### UL Increases the mRNA Levels of H1, H2 and H3 Receptors in the Ipsi-lesional Flocculus and MVN

**Figure 2:**
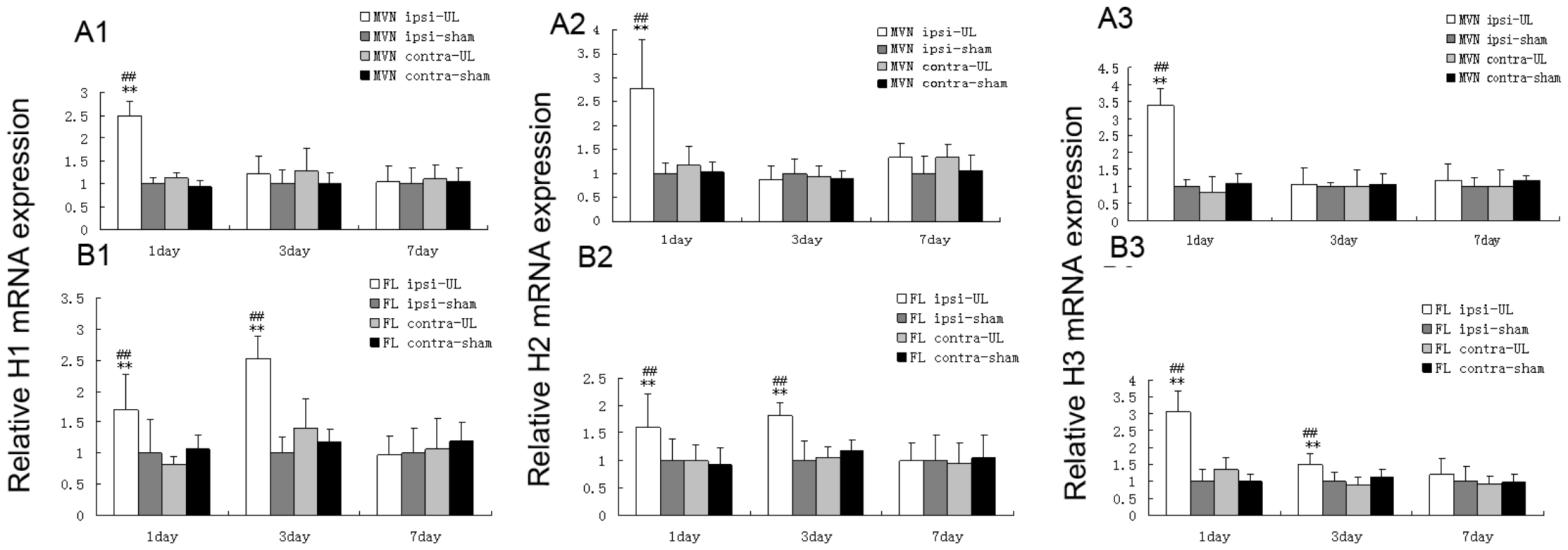
The mRNA expression of H1, H2 and H3 receptors. Quantitative analysis of the H1, H2 and H3 receptors mRNA in the MVN (A1–3) and the flocculus (B1–3) on the 1st, 3rd and 7th day following UL or sham operation. The H1, H2 and H3 receptors mRNA levels significantly increased in the ipsi-lesional MVN on the 1st day following UL, and H1, H2 and H3 receptors mRNA levels increased in ipsi-lesional flocculus on the 1st and 3rd day following UL as well. Columns represent means±SEM of 6 rats per group. **P<0.01 vs. sham; ^##^ *P*<0.01 vs. contra. FL, flocculus; MVN, medial vestibular nucleus.

On the 1st and the 3rd day after UL, the mRNA expression of H1 (Fig. 2B1), H2 (Fig. 2B2) and H3 (Fig. 2B3) receptors on the ipsi-lesional flocculus was significantly increased, compared with that on the contra-lesional flocculus and with that of the control group as well. However, on the 7th day, the differences between them became minimal. Please note, the contra-lesional side and the sham controls have no related changes of the mRNA expression (Fig. 2B1–3). Moreover, in the ipsi-lesional MVN, the mRNA levels of the H1 (Fig. 2A1), H2 (Fig. 2A2) and H3 (Fig. 2A3) receptors underwent a large and significant increase on the 1st day after UL compared with either the contra-lesional side or the sham controls. On the other side, on the 3rd and the 7th day after UL, the difference between them vanished. Similarly, the contra-lesional side and the sham controls did not show any obvious change in the data (Fig. 2A1–3).

### UL Increases the Protein Expression of H1, H2 and H3 Receptors in the Ipsi-lesional Flocculus and MVN

**Figure 3:**
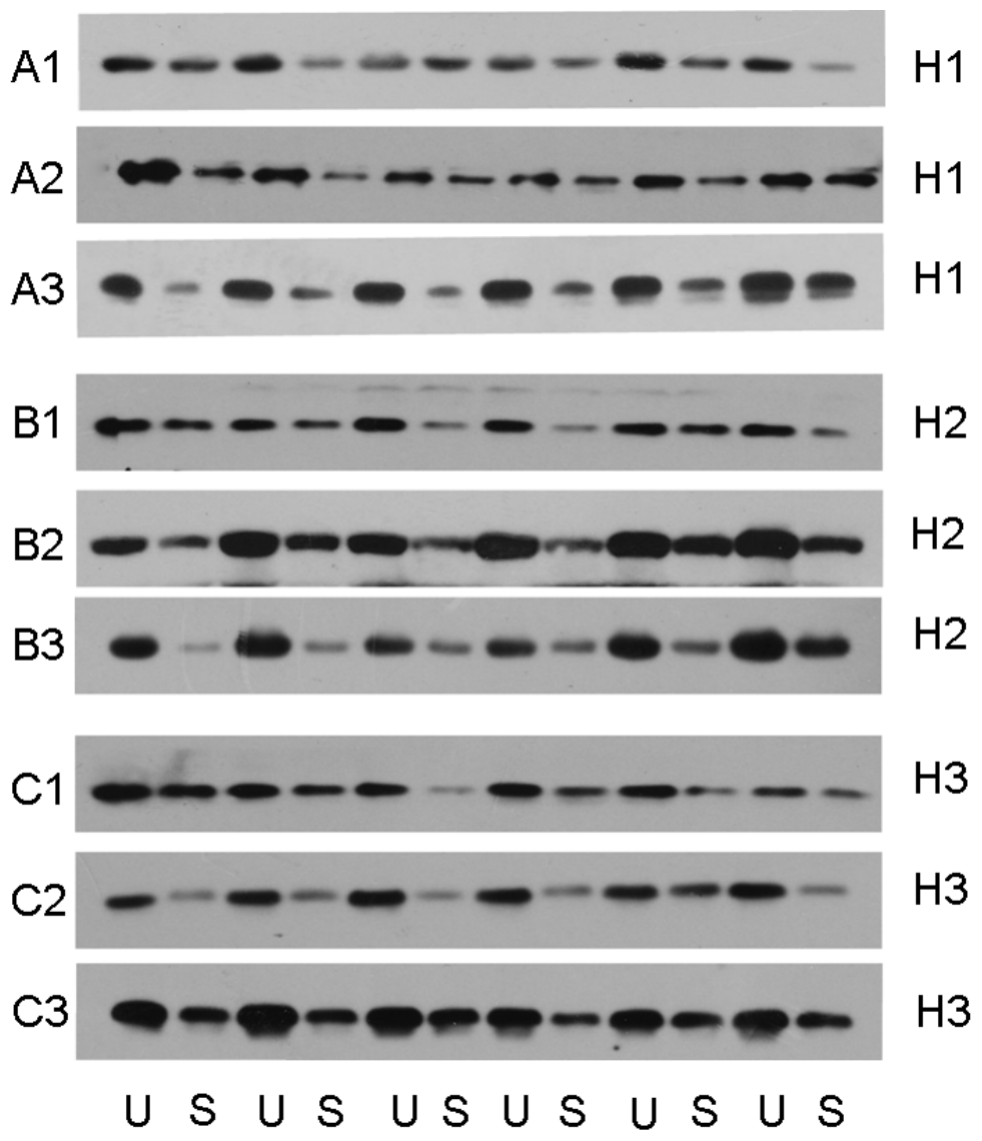
Immunoblot of histamine receptors in the flocculus and the MVN. Samples assayed are shown as follows: H1 (A1, 1 day post-UL in the MVN; A2, 1 day post-UL in the flocculus; A3 3 day post-UL in the flocculus) H2 (B1, 1 day post-UL in the MVN; B2 1 day post-UL in the flocculus; B3 3 day post-UL in the flocculus) H3 (C1, 1 day post-UL in the MVN; C2 1 day post-UL in the flocculus; C3 3 day post-UL in the flocculus). U, unilateral labyrinthectomy; S, Sham operation.

**Figure 4:**
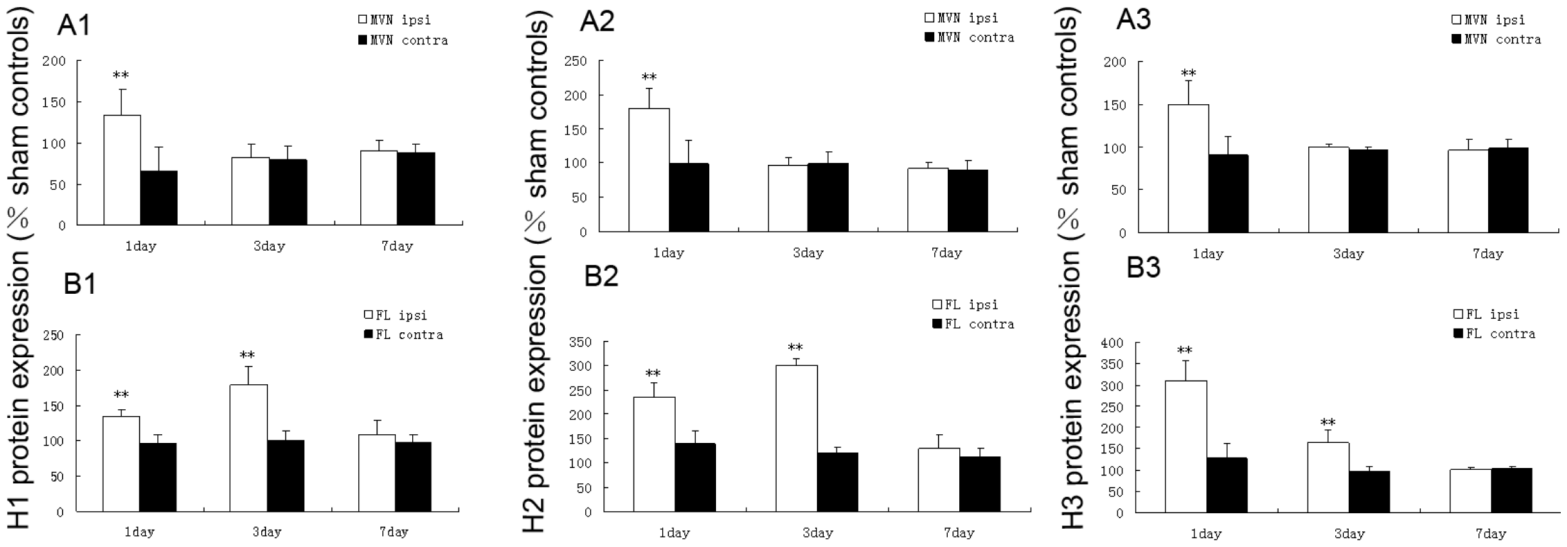
H1, H2 and H3 receptors protein expression. The expression is expressed as a percentage of sham controls, in the MVN (A1–3) and flocculus (B1–3) on the 1st, 3rd and 7th day following UL. The protein levels of the H1, H2 and H3 receptors significantly increased in the ipsi-lesional MVN on the 1st day following UL, and protein levels of the H1, H2 and H3 receptors increased in ipsi-lesional flocculus on the 1st and 3rd day following UL as well. Columns represent means±SEM of 6 rats per group. **P<0.01 vs. sham. ipsi, ipsi-lesional side, contra, contra-lesional side.

Similar to mRNA expression of H1, H2 and H3 receptors, the expression of corresponding H1 (Fig. 3A2–3 and Fig.4 B1), H2 (Fig. 3B2–3 and Fig. 4B2) and H3 (Fig. 3C2–3 and Fig. 4B3) receptors proteins in the ipsi-lesional flocculus was increased significantly, compared with both that on the contra-lesional side and in the control group on the 1st and the 3rd day after UL. On the 7th day, the difference between them was minimal, while the contra-lesional side and the sham controls have no related changes of the protein expression (Fig. 4B1–3). Additionally, in the ipsi-lesional MVN, the protein levels of the H1 (Fig. 3A1 and Fig. 4A1), H2 (Fig. 3B1 and Fig. 4A2) and H3 (Fig. 3C1 and Fig. 4A3) receptors were significantly increased on the 1st day after UL compared with both that on the contra-lesional side and in the control group, while on the 3rd and 7th day after UL, the difference was minimal. Similarly, the contra-lesional side and the sham controls did not show any obvious change in the data (Fig. 4A1–3).

### UL Induces the Asymmetrical Distribution of H1, H2 and H3 Receptors in Bilateral Flocculus and MVN

**Figure 5:**
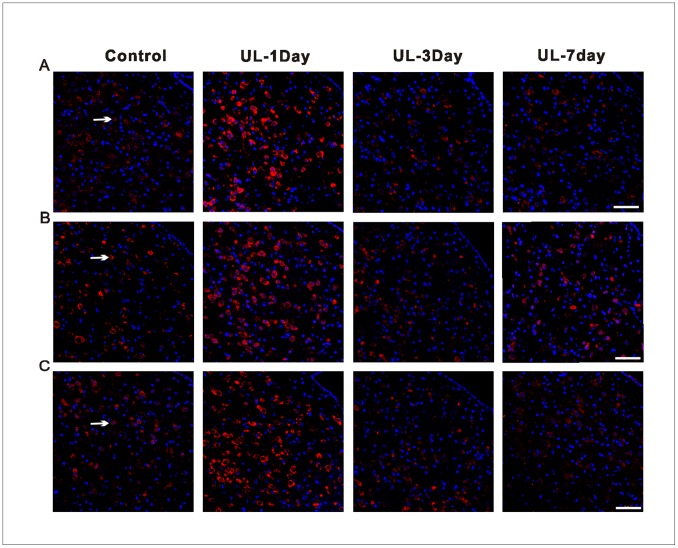
Localization and distribution of histamine receptors in the MVN. Representative confocal images showing the localization and distribution of H1 (A), H2 (B) and H3 (C) receptors in the same area of the ipsi-lesional MVN following UL or sham operation at different postoperative days. Arrows show the H1-, H2- and H3 receptors -positive neurons. UL-1day, 1 day after UL; UL-3day, 3 day after UL, UL-7day, 7 day after UL; Control, with intact labyrinths; Calibration bar = 50 µm.

**Figure 6:**
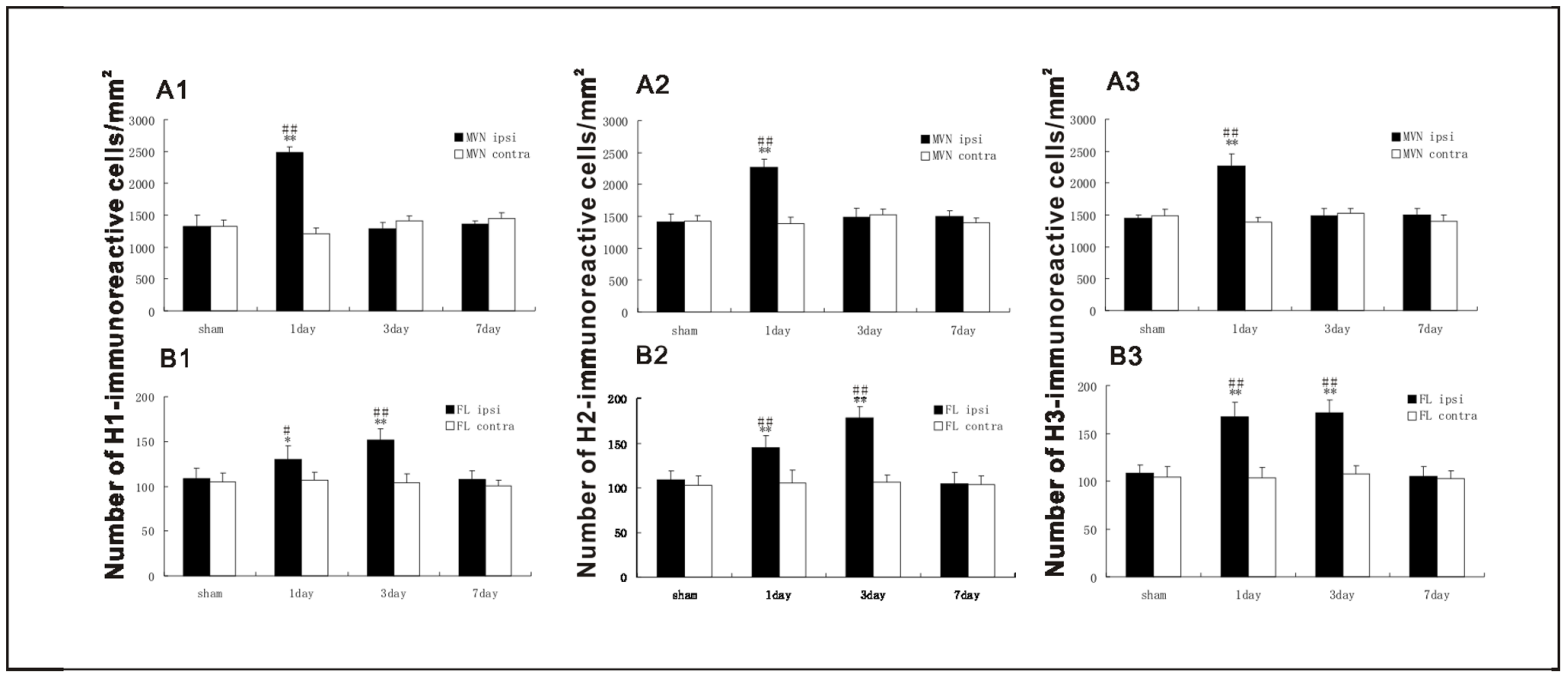
Changes in the number of H1, H2 and H3 receptors-positive neurons in the MVN and the ﬂocculus after UL or sham operation. ipsi, ipsi-lesional side, contra, contra-lesional side. Number of H1, H2 and H3 receptors-positive neurons in each corresponding region of the visual system was expressed per unit area (mm^2^). **P<0.01 vs. sham; ^##^ *P*<0.01 vs. contra; *P<0.05 vs. sham; ^#^ *P*<0.05 vs. contra.

**Figure 7:**
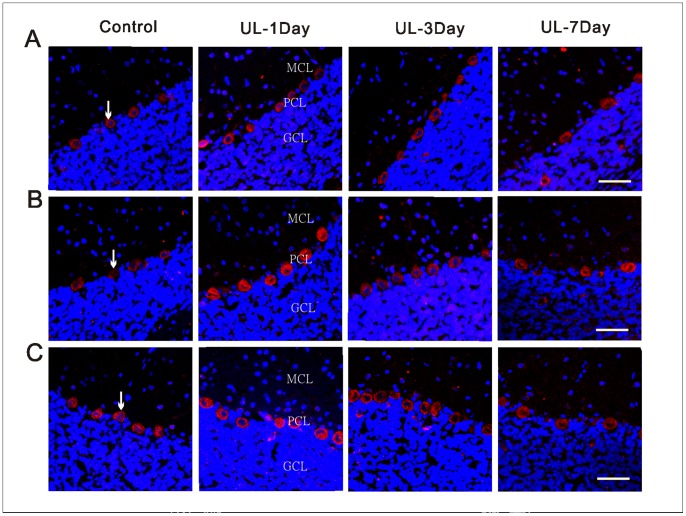
Localization and distribution of histamine receptors in the flocculus. Representative confocal images showing the expression and localization of the H1 (A), H2 (B) and H3 (C) receptors in the same area of the ipsi-lesional flocculus following UL or sham operation at different post-operative days. Arrows show the H1-, H2- and H3 receptors -positive neurons. UL-1day, 1 day after UL; UL-3day, 3 day after UL; UL-7day, 7 day after UL; Control, with intact labyrinths; GCL, granule cell layer; PCL, Purkinje cell layer; MCL, molecular cell layer. Calibration bar = 40 µm.

The H1, H2 and H3 receptors were detected by the immunohistochemistry technique on both the MVN and the flocculus. The H1 (Fig. 5A), H2 (Fig. 5B) and H3 (Fig. 5C) receptors of the MVN neurons were immunostained in the membranes and cytoplasm. In the control groups, there were no significant differences in the number of H1, H2 and H3 receptors-positive neurons in the MVN and flocculus between the four sections (Fig. S2). The H1, H2 and H3 receptors-positive neurons were symmetrically distributed between bilateral MVN. With the UL groups, at the 1st day, the H1, H2 and H3 receptors-positive neurons distributed asymmetrically between bilateral MVN. That is, the number of H1 (Fig. 6A1), H2 (Fig. 6A2) and H3 (Fig. 6A3) receptors-positive neurons was significantly increased in the ipsi-lesional MVN compared with either that in the contra-lesional side or that in the control groups. While at the 3rd and 7th day the asymmetrical distribution is minimal. In the flocculus, the H1 (Fig. 7A), H2 (Fig. 7B) and H3 (Fig. 7C) receptors-positive neurons were concentrated in the molecular and Purkinje cell layers, especially in the Purkinje cell layers. The H1, H2 and H3 receptors-positive neurons were symmetrically distributed between bilateral flocculus in control groups. At the 1st and 3rd day after UL, the H1, H2 and H3 receptors-positive neurons distributed asymmetrically between the bilateral flocculus. That is, the number of H1 (Fig. 6B1), H2 (Fig. 6B2) and H3 (Fig. 6B3) receptors-positive neurons was significantly increased in the ipsi-lesional ﬂocculus compared with either that in the contra-lesional side or that in the control groups. While at the 7th day, this asymmetrical distribution disappeared again.

## Discussion

Here we demonstrated the spatiotemporal dynamics of H1, H2 and H3 receptors induction in the MVN and the flocculus on the 1st, 3rd and 7th day after UL. The present study provides the quantitative and systematic data regarding H1, H2 and H3 receptors mRNA and protein levels in the cerebello-vestibular pathway during the process of vestibular compensation. After the UL treatment with rats, there is an increase in both mRNA and protein levels of H1, H2 and H3 receptors in the ipsi-lesional flocculus and the MVN. To the best of our knowledge, this study is the first molecular investigation of histamine receptors expression in the cerebello-vestibular pathway following UL in rats.

Compared with either the contra-lesional side or with the sham controls, the ipsi-lesional flocculus had significantly higher expression of the H1, H2 and H3 receptors on the 1st and 3rd day after UL, while such enhancement disappeared at the 7th day after UL. The cerebello-vestibular pathway, originated from Purkinje cells of the anterior cerebellar vermis, mediates monosynaptic inhibition of the vestibular nucleus neurons. [11], [45] As the major inhibitory neurons, Purkinje cells release GABA, and GABA could be one of the inhibitory neurotransmitters in the cerebello-vestibular pathway. [46] During the process of vestibular compensation, MVN neurons on both sides of the brainstem develop the rebalance of their resting activity, and the flocculus plays an important role in the process. It has been proposed that ipsi-lesional flocculectomy prevented the compensatory increase of intrinsic excitability in the de-afferented MVN neurons after UL [47], [48].

In addition to changes in the intrinsic membrane excitability of bilateral MVN neurons, there is a concurrent, rapid downregulation of the functional efficacy of GABA and glycine receptor expression in the ipsi-lesional MVN after UL. [49]–[52] Previous studies have indicated that histaminergic could inhibit the release of GABA at Purkinje cell synapses in two ways: 1) through a direct action on presynaptic H3 receptor (presumably located on GABAergic terminals), and 2) through a novel, indirect pathway that involved the increased releasing of glycine by activation of postsynaptic H1/H2 receptor (presumably on glycinergic neurons). [12] In our study, the upregulation of H1, H2 and H3 receptors in ipsi-lesional flocculus might abate the inhibitory GABAergic signals from the ipsi-lesional flocculus to the ipsi-lesional MVN neurons, which consequently rebalanced the resting activity in bilateral MVN neurons after UL.

The expression of H1, H2 and H3 receptors in the ipsi-lesional MVN was increased on the 1st day after UL but returned to the level of the control groups on the 3rd and the 7th day after UL. The changes in histamine receptors expression observed in our study are consistent with the temporal changes in the early static vestibular manifestations and the changes in MVN neurons metabolic activity during vestibular compensation. [2], [33] These results are also consistent with Lozada’s data, [28] who found an increase in the mRNA levels of H3 receptor isoforms in MVN 24 h after UL by performing in situ hybridization in rats. While on the 2nd and 7th day post-UL, the mRNA expression returned to control levels. However, using autoradiography, Tighilet *et al* [*27*] reported that H3 receptor binding levels were downregulated in the MVN at the 7th day following unilateral vestibular neurectomy in cats, such discrepancy between the different data might be due to differences in experimental performances, operation methods and the animal species tested.

Immediately after UL, the resting activity of ipsi-lesional MVN neurons is largely abolished, while contra-lesional MVN neurons are hyperactive. [33] Ipsi-lesional MVN neurons silencing is mediated by the commissural inhibition system, which links each MVN to its contralateral counterpart. [52] The recovery of resting discharge in the ipsi-lesional MVN neurons and the rebalancing of the resting activity of the bilateral MVN neurons after UL may be achieved by compensatory change in the gains of the commissural inhibition system. [52] This compensatory change after UL is believed to be accompanied and exacerbated by an imbalance in GABA receptor interactions across the bilateral MVN neurons. [53] Therefore, the dynamic regulation of GABAergic inhibitory efficacy potentially rebalances the bilateral excitability of MVN neurons after UL [54].

It has been shown that histamine inhibits the stimulus-evoked release of GABA in the MVN, both through a direct activation of pre-synaptic H3 receptor on GABAergic synaptic terminals, and through an indirect pathway that involves the increased release of glycine by H1/H2 receptor. [12] In response to the compensatory change of commissural inhibition, there is a marked down-regulation of GABA receptor efficacy in the ipsi-lesional MVN neurons after UL during vestibular compensation *in viv*o. [52] Thus it is possible that endogenous-release of H1, H2 and H3 receptors in the ipsi-lesional MVN may modulate the GABA release in the commissural system after UL. As a result, this over expression of histamine receptors promotes the disinhibition of neurons in the ipsi-lesional MVN neurons, and induces the rebalance of the resting activity of bilateral MVN neurons.

In our study, the expression of histamine receptors in bilateral MVN were compared with each other and with the sham operation controls, and that in bilateral flocculus were compared with each other and with the sham operation controls as well. These experiments were performed in the same situation during the same time for each run. Therefore, these results were stable and confirmable. While the histamine receptors in the contra-lesional MVN and contra-lesional flocculus do not change overtime. It is possible that function of histamine receptors in the contra-lesional MVN and flocculus may change during vestibular compensation as a result of modifications in affinity or efficacy.

**Figure 8:**
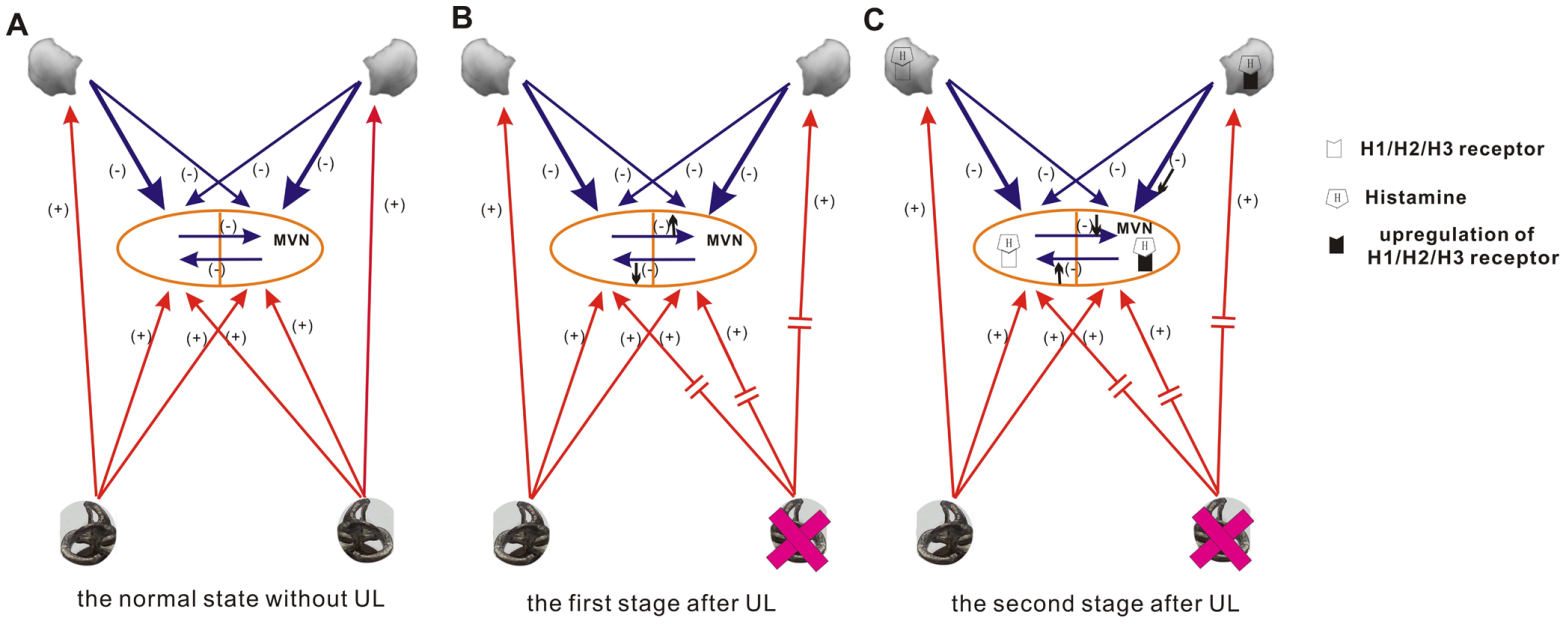
Schematic diagram of the changes in histamine receptors expression in the flocclar-MVN pathways following UL. A. the normal state without UL: The resting activity of bilateral MVN neurons remains balance because of the inhibitory effects of flocculus and commissural inhibition system and the excitable effects of periphery vestibular organs. B The first stage after UL: Immediately after UL, due to the inhibitory effects of flocculus and commissural inhibition system, the resting activity of ipsi-lesional MVN neurons is largely abolished, while contra-lesional MVN neurons are hyperactive. C The second stage after UL: Upregulation of histamine receptors (H1, H2, H3) in ipsi-lesional MVN and flocculus may reduce the inhibitory effects of flocculus and commissural inhibition system by regulation GABA, so as to rebalance the resting activity of bilateral MVN neurons. MVN, medial vestibular nucleus; UL, unilateral labyrinthectomy.

In conclusion, we investigated the changes of both the mRNA and the protein expression of H1, H2 and H3 receptors in the MVN and the flocculus at the 1st, the 3rd and the 7th day after UL. Our results have demonstrated that the H1, H2 and H3 receptors are upregulated in both the ipsi-lesional MVN and the ipsi-lesional flocculus in the early stage (within three days) during vestibular compensation, which indicated that histamine receptors in the MVN and flocculus may play roles in the early stage (within three days) of vestibular compensation. In addition, other neurotransmitters in the central nervous system, or histamine receptors in other regions of the central nervous system may participate in the later stage of vestibular compensation. Upregulation of histamine receptors in ipsi-lesional MVN and ipsi-lesional flocculus may reduce the inhibitory effects of flocculus and commissural inhibition system by regulation GABA, so as to rebalance the resting activity of bilateral MVN neurons (Fig. 8C). The histamine play an important role as a neurotransmitter and neuromodulator in the cerebello-vestibular pathway during the process of vestibular compensation. Histaminergic modulation of neurotransmitter release in the cerebello-vestibular pathway is important in vestibular synaptic plasticity and behavioural recovery after UL. However, such aspect is beyond the scope of this study and should be further explored.

## Supporting Information

Figure S1 **Immunoblot of histamine receptors in the MVN and the flocculus after mechanical UL.** Samples assayed are shown as follows: A, 1 day post-mechanical UL in the MVN; B, 1 day post-mechanical UL in the flocculus; C, 3 day post- mechanical UL in the flocculus U, unilateral labyrinthectomy; S, sham operation. The protein levels of the H1, H2 and H3 receptors significantly increased in the ipsi-lesional MVN on the 1st day following the mechanical UL, and protein levels of the H1, H2 and H3 receptors increased in ipsi-lesional flocculus on the 1st and 3rd day following mechanical UL as well.
(TIF)

Figure S2 **Histograms of the neurons counting of H1, H2 and H3 receptors-positive neurons in the rostral, middle and caudal part of the MVN and the ﬂocculus in control groups.** Number of H1, H2 and H3 receptors-positive neurons in each corresponding region of the visual system was expressed per unit area (mm^2^).
(TIF)
