# Supplementary figures and images for: Changes in Histamine Receptors (H1, H2, and H3) Expression in Rat Medial Vestibular Nucleus and Flocculus after Unilateral Labyrinthectomy: Histamine Receptors in Vestibular Compensation
Source: PLoS One. 2013 Jun 19;8(6):e66684. doi: 10.1371/journal.pone.0066684 (PMC3686684; doi:10.1371/journal.pone.0066684)

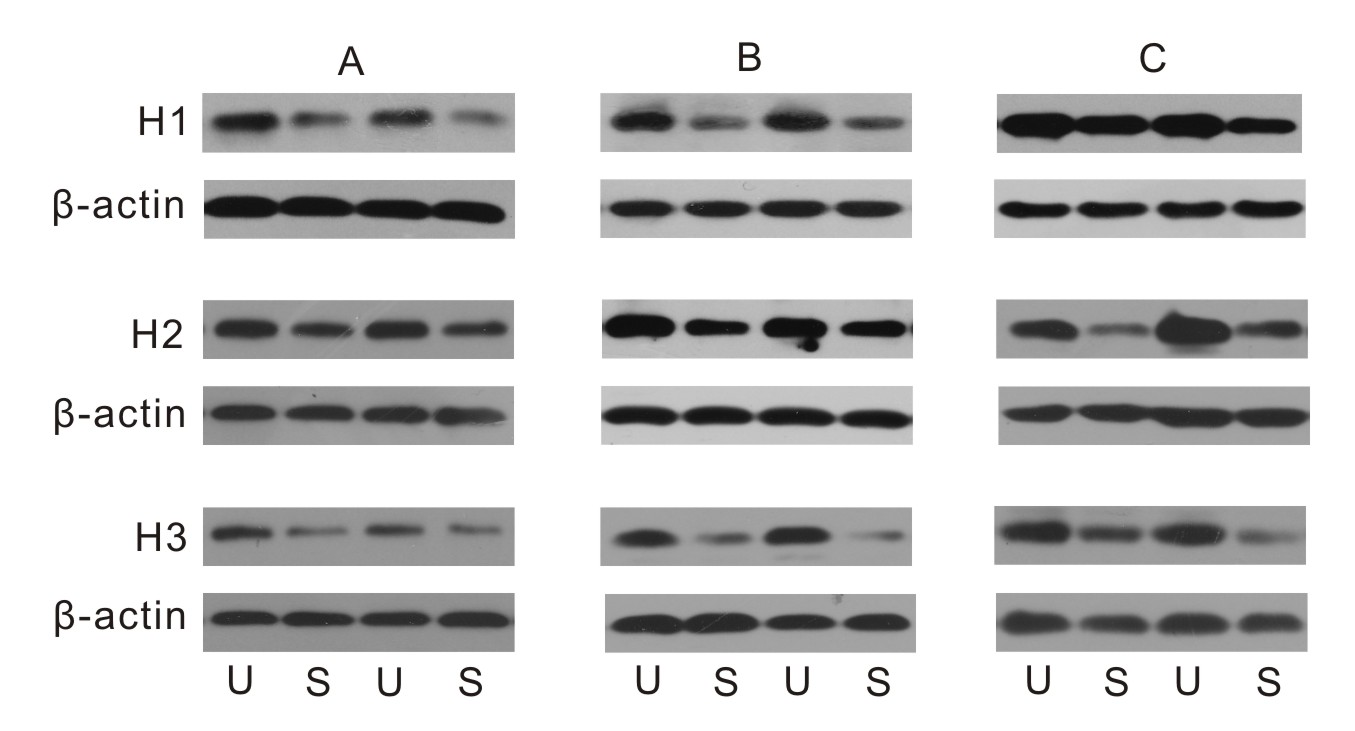

Supplement: Figure S1 — Immunoblot of histamine receptors in the MVN and the flocculus after mechanical UL. Samples assayed are shown as follows: A, 1 day post-mechanical UL in the MVN; B, 1 day post-mechanical UL in the flocculus; C, 3 day post- mechanical UL in the flocculus U, unilateral labyrinthectomy; S, sham operation. The protein levels of the H1, H2 and H3 receptors significantly increased in the ipsi-lesional MVN on the 1st day following the mechanical UL, and protein levels of the H1, H2 and H3 receptors increased in ipsi-lesional flocculus on the 1st and 3rd day following mechanical UL as well. (TIF) [file pone.0066684.s001.tif]

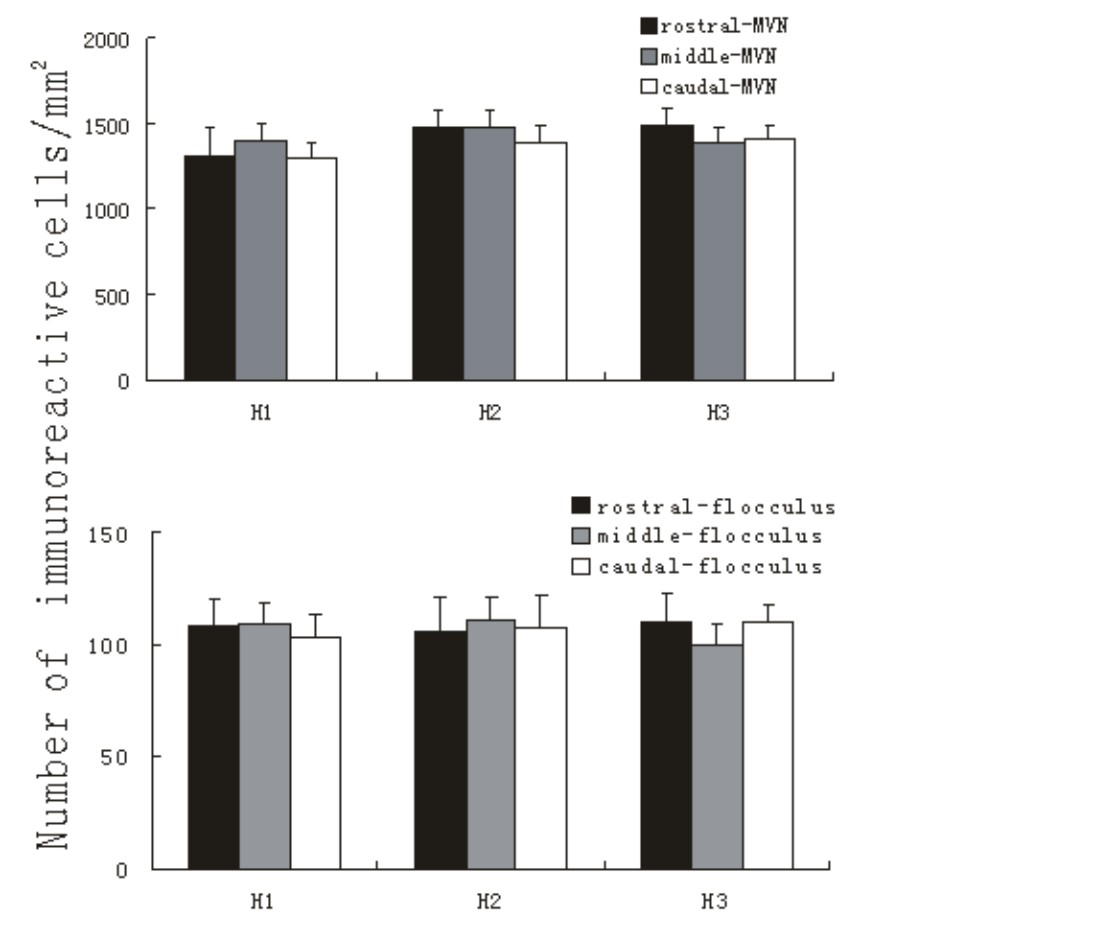

Supplement: Figure S2 — Histograms of the neurons counting of H1, H2 and H3 receptors-positive neurons in the rostral, middle and caudal part of the MVN and the ﬂocculus in control groups. Number of H1, H2 and H3 receptors-positive neurons in each corresponding region of the visual system was expressed per unit area (mm2). (TIF) [file pone.0066684.s002.tif]
